# Eco-friendly zinc oxide nanoparticle biosynthesis powered by probiotic bacteria

**DOI:** 10.1007/s00253-024-13355-4

**Published:** 2025-01-29

**Authors:** Ahmed Issa AL-Tameemi, Mas Jaffri Masarudin, Raha Abdul Rahim, Rachel Mizzi, Verlaine J. Timms, Nurulfiza mat Isa, Brett A. Neilan

**Affiliations:** 1 School of Environmental and Life Sciences, The University of Newcastle, Callaghan, NSW 2308 Australia; 2 Department of Cell and Molecular Biology, Faculty of Biotechnology and Biomolecular Sciences, Universiti Putra Malaysia, 43400 UPM Serdang, Selangor Malaysia; 3 Institute of Bioscience, Universiti Putra Malaysia, 43400 UPM Serdang, Selangor Malaysia; 4 College of Dentistry, Al-Iraqia University, 10053 Al Adhamiya, Baghdad, Iraq

**Keywords:** Probiotic bacteria, Biosynthesis, ZnO NPs, Antimicrobial activity

## Abstract

**Abstract:**

The rapid advancement of nanotechnology, particularly in the realm of pharmaceutical sciences, has significantly transformed the potential for treating life-threatening diseases. A pivotal aspect of this evolution is the emergence of “green nanotechnology,” which emphasizes the environmentally sustainable synthesis of raw materials through biological processes. This review focuses on the biological synthesis and application of zinc oxide (ZnO) nanoparticles (NPs) from probiotic bacteria, particularly those sourced from wastewater. Microorganisms from wastewater tolerate harmful elements and enzymatically convert toxic heavy metals into eco-friendly materials. These probiotic bacteria are instrumental in the synthesis of ZnO NPs and exhibit remarkable antimicrobial properties with diverse industrial applications. As the challenge of drug-resistant pathogens escalates, innovative strategies for combating microbial infections are essential. This review explores the intersection of nanotechnology, microbiology, and antibacterial resistance, highlighting the importance of selecting suitable probiotic bacteria for synthesizing ZnO NPs with potent antibacterial activity. Additionally, the review addresses the biofunctionalization of NPs and their applications in environmental remediation and therapeutic innovations, including wound healing, antibacterial, and anticancer treatments. Eco-friendly NP synthesis relies on the identification of these suitable microbial “nano-factories.” Targeting probiotic bacteria from wastewater can uncover new microbial NP synthesis capabilities, advancing environmentally friendly NP production methods.

**Key points:**

*• Innovative strategies are needed to combat drug-resistant pathogens like MRSA.*

*• Wastewater-derived probiotic bacteria are an eco-friendly method for ZnO synthesis.*

*• ZnO NPs show significant antimicrobial activity against various pathogens.*

## Introduction

ZnO NPs have garnered significant attention due to their diverse applications across various industries, including medicine, environmental remediation, and electronics (Goswami et al. 2024). Initially utilized in the rubber industry for waterproofing and enhancing the durability of composite materials (Wang et al. 2023b), ZnO NPs are now increasingly recognized for their strong UV absorption properties, making them valuable in cosmetics and sunscreen formulations (Chauhan et al. 2022). Their effectiveness in blocking UV radiation is influenced by their size and morphology, which allows them to convert harmful UV rays into harmless infrared light (Sasani Ghamsari et al. 2017). Additionally, ZnO NPs impart antibacterial and deodorizing properties when incorporated into textile fabrics (Tamilvanan and Ramadoss 2024). Beyond these applications, ZnO NPs are being explored for their biomedical potential, including anticancer therapies, drug delivery systems, and wound healing (Moalwi et al. 2024). Their cost-effectiveness and lower toxicity compared to other metal oxide NPs further enhance their appeal in these fields (Moalwi et al. 2024).

The synthesis of ZnO NPs is critical to their application, and various methods exist, including physical, chemical, and biological routes (Ashraf et al. 2023). Traditional synthesis methods often involve toxic chemicals and high energy consumption, raising environmental concerns. In contrast, biological synthesis methods offer eco-friendly and cost-efficient alternatives, utilizing microorganisms such as bacteria, yeast, and fungi (Sachin and Karn 2021). The selection of suitable microbial candidates is essential for optimizing NP production, as different microorganisms exhibit varying metabolic processes and enzyme activities (Mohd Yusof et al. 2019).

Wastewater treatment plants (WWTPs) represent a significant reservoir of diverse microorganisms, including potential probiotic species capable of synthesizing NPs (Al-Tameemi et al. 2023b). The unique microbial composition found in WWTPs, derived from industrial and domestic waste (Ariyadasa et al. 2023), provides an opportunity to explore the synthesis of ZnO NPs using these microorganisms (Al-Tameemi et al. 2023a). Wastewater contains microorganism with high tolerance to toxic substances and enzymatic reduction ability, making it a rich source of species that can synthesize NPs. This characteristic is particularly advantageous, as these bacteria can thrive in harsh environments and possess the necessary enzymatic pathways to facilitate the reduction of metal ions into NPs (Al-Tameemi et al. 2023b). Targeting probiotic bacteria from wastewater presents a dual approach to the discovery of new microbial NP synthesis capabilities. This pioneering research will drive advancements in environmentally conscious NP synthesis.

This review distinguishes itself by comprehensively analyzing the biosynthesis of ZnO NPs using zinc-resistant probiotic bacteria, especially sourced from wastewater. Unlike previous reviews that focused on challenges in metal NP synthesis, this study offers a novel approach that addresses development of effective antibacterial agents and promoting eco-friendly NP synthesis practices (Mohd Yusof et al. 2019; Altammar 2023). Altammar (2023) highlighted difficulties in controlling the size and shape of metal NPs and minimizing their environmental impact, particularly the toxicity of silver NPs to aquatic life. In contrast, our research focuses on the biosynthesis of ZnO NPs using environmentally friendly bacterial strains, which mitigates toxicity concerns and improves control over particle characteristics through biological mechanisms.

## Bacterial genera considered as probiotic

*Lactobacillaceae* are gram-positive, non-sporulating, and facultative or strictly anaerobic with coccoid or rod-shaped cells (Walter and O'Toole 2023). These bacteria inhabit nutrient-rich environments found in the human gut, food, plants, feed, wastewater, invertebrates, and vertebrate animals (Walter and O'Toole 2023; Bhakta et al. 2012). It is widely acknowledged that *Lactobacillaceae* genera such as *Latilactobacillus sakei*, *L. planatrum*, *Pediococcus pentosaceus* and *Enterococcus faecium*, *Weissella cibaria*, and *W. confusa* are beneficial probiotics for humans and animals (Ahmed et al. 2022; Mushtaq et al. 2021). Ahmed et al. (2022) reported that *Weissella* strains such as *W. cibaria* and *W. confusa* have probiotic properties to improve oral health, skincare, anticancer, inflammation, and obesity (Ahmed et al. 2022). Korean researchers market these strains under the names oraCMS1 and oraCMU, which both promote gum health and bad breath control (Kang et al. 2020). Yeong et al. (2020) mentioned that the *Weissella* production of exopolysaccharides (EPSs), antimicrobial compounds, organic acids, acetate, ethanol, and volatile compounds enhances the physicochemical properties, flavor, and texture of a variety of food products. Together, these uses of the *Lactobacillaceae* demonstrate their probiotic potential, making them promising candidates for enhancing animal and human health.

In addition to their probiotic properties, *Lactobacillaceae* can act as a biosorbent for the removal of heavy metals from the environment. Numerous *Lactobacillaceae* species, including *Bacillus*, *Lactobacillus*, *Lactococcus*, *Enterococcus*, *Bifidobacterium*, *Pediococcus*, *Propionibacterium*, *Streptococcus*, and *Weissella*, have displayed the ability to detoxify heavy metals such as arsenic, cadmium, chromium, mercury, lead from food, the body, wastewater, and the environment (Li et al. 2021; Massoud and Zoghi 2022). The resistance of bacteria to metal ions could be a crucial factor in the formation of NPs, especially when metal stresses are present (Mohd Yusof et al. 2020b). Furthermore, probiotic bacteria that are resistant to heavy metals produce enzymes that function as stabilizing and reducing agents to synthesize NPs. For example, ZnO NPs have been synthesized from zinc-resistant *L. plantarum* using both supernatants and cell biomass (Mohd Yusof et al. 2020a). *Lactobacilli*, especially *L. plantarum*, have been widely studied for their probiotic properties and technological potential in the feed and food industries. As well as being capable of absorbing and tolerating heavy metal ions, they also have potential for environmental remediation, as demonstrated by their ability to synthesize metal NPs under stress (Zommara et al. 2023; Wang et al. 2023a; Loi et al. 2023). Likewise, *Pediococcus* species show probiotic potential and resistance to metal ions, opening up possibilities for bioremediation and health promotion. *Pediococcus* NP synthesis further expands their potential contributions to environmental detoxification and nanotechnology efforts (Todorov et al. 2023; Massoud and Zoghi 2022). *Weissella* sp., defined by their probiotic and antimicrobial properties, have emerged as potential candidates for health and well-being (Kim et al. 2023).

Notably, *Weissella*’s capacity to resist zinc ions and produce ZnO NPs as antibacterial agents has received less attention. In our previous study, zinc-resistant strains, *W. cibaria* UPM22MT06 and *W. confusa* UPM22MT04, demonstrated the ability to simultaneously resist zinc and produce ZnO NPs, highlighting their potential in both metal ion resistance and NP synthesis (Al-Tameemi et al. 2023a, 2023b).

In short, *Lactobacillaceae* species exhibit remarkable versatility and potential across various domains. Ongoing research continues to unveil their capabilities, offering new avenues for biotechnological advancements. Exploring these characteristically potential probiotics from heavy metal-polluted environmental samples represents a promising biotechnological approach for the future.

## Nanotechnology

Nanotechnology, initially defined by Japanese Professor Taniguchi (1974), encompasses the manipulation and transformation of materials at the atomic or molecular level. This involves processes such as separation, consolidation, and deformation. The concept was later introduced by Drexler (1981) and has since branched into diverse disciplines, including biology, chemistry, physics, and materials science (Manjul and Pant 2013). The appeal of nanotechnology lies in its unique capacity to engineer materials at the nanoscale, typically within the range of up to 100 nm (Mustapha et al. 2020). These NPs, due to their tiny dimensions and high surface area-to-volume ratio, exhibit distinctive optical, mechanical, catalytic, and biological characteristics that surpass those of their bulk counterparts (Marimuthu et al. 2020).

**Fig. 1:**
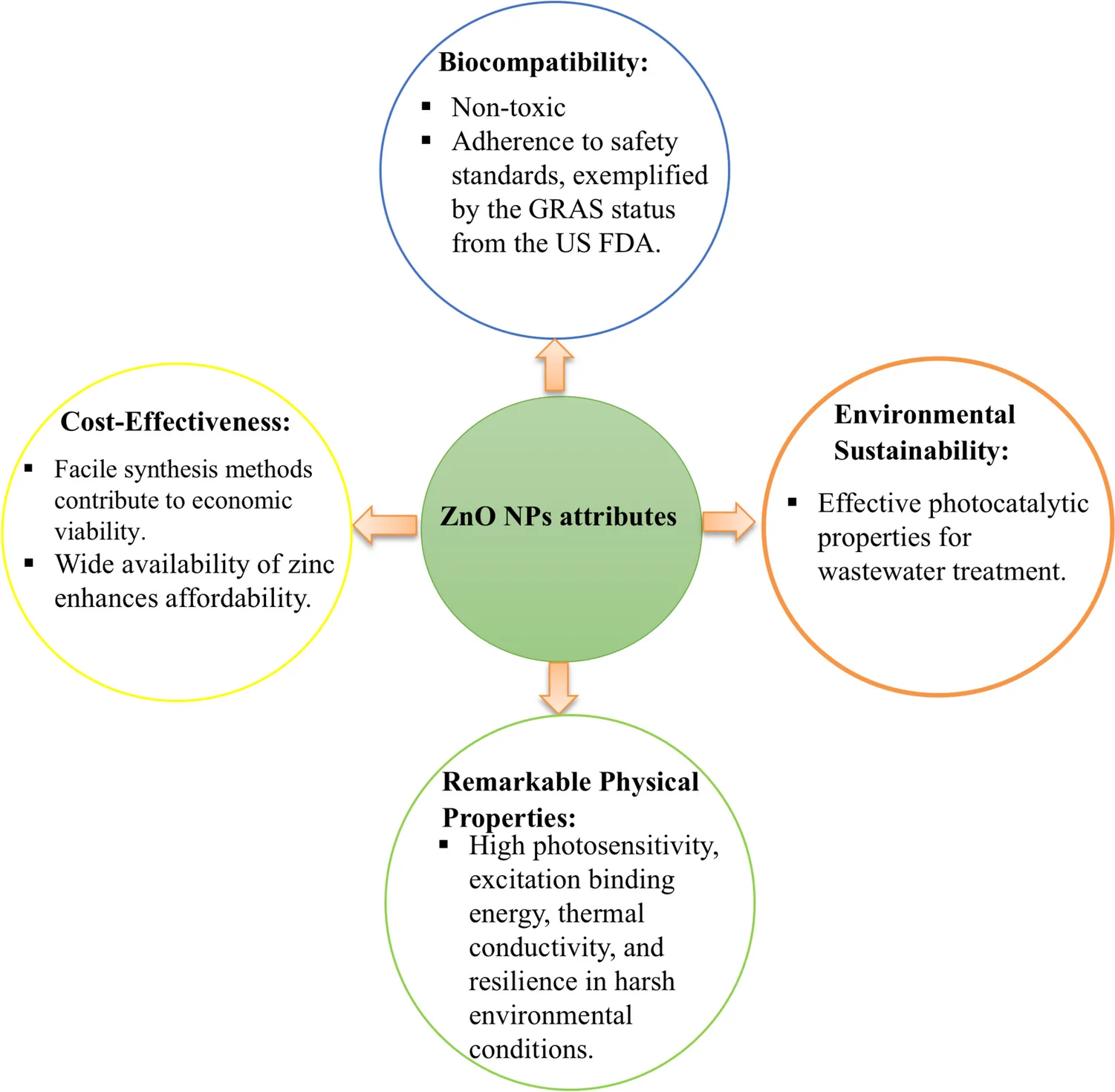
A visual representation of the various attributes associated with ZnO NPs

Among various types of metal oxide NPs, such as titanium dioxide (TiO_2_), indium (III) oxide (In_2_O_3_), ZnO, tin (IV) oxide (SnO_2_), and silicon dioxide (SiO_2_) (Piccinno et al. 2012; Anvarinezhad et al. 2020), ZnO NPs have emerged as a subject of significant research interest. This heightened attention is attributable to their remarkable properties, such as biocompatibility, environmental sustainability, cost-effectiveness, facile synthesis, high photosensitivity, substantial excitation binding energy, superior thermal conductivity, and resilience in harsh environmental conditions as shown in Fig. 1 (Mohammadi and Ghasemi 2018). In addition to these advantages, ZnO NPs hold substantial utility across a wide spectrum of applications, including electronics, optics, food packaging, cosmetic products, petroleum industries, pharmaceuticals, and agriculture. Notably, ZnO NPs play a pivotal role in various biomedical applications already, serving as anticancer, antimicrobial, anti-inflammatory and wound healing agents, antioxidants, and drug delivery vehicles (Nandhini et al. 2024).

ZnO NPs exhibit highly effective photocatalytic properties for wastewater treatment, offering a promising approach when addressing challenges related to environmental pollution (Hussain et al. 2024). It is noteworthy that ZnO has been granted GRAS status by the US FDA emphasizing its non-toxic nature (Eren et al. 2024). This aspect underscores the potential for safe and widespread utilization of ZnO NPs in various scientific and industrial endeavors.

## Synthesis of NPs

**Fig. 2:**
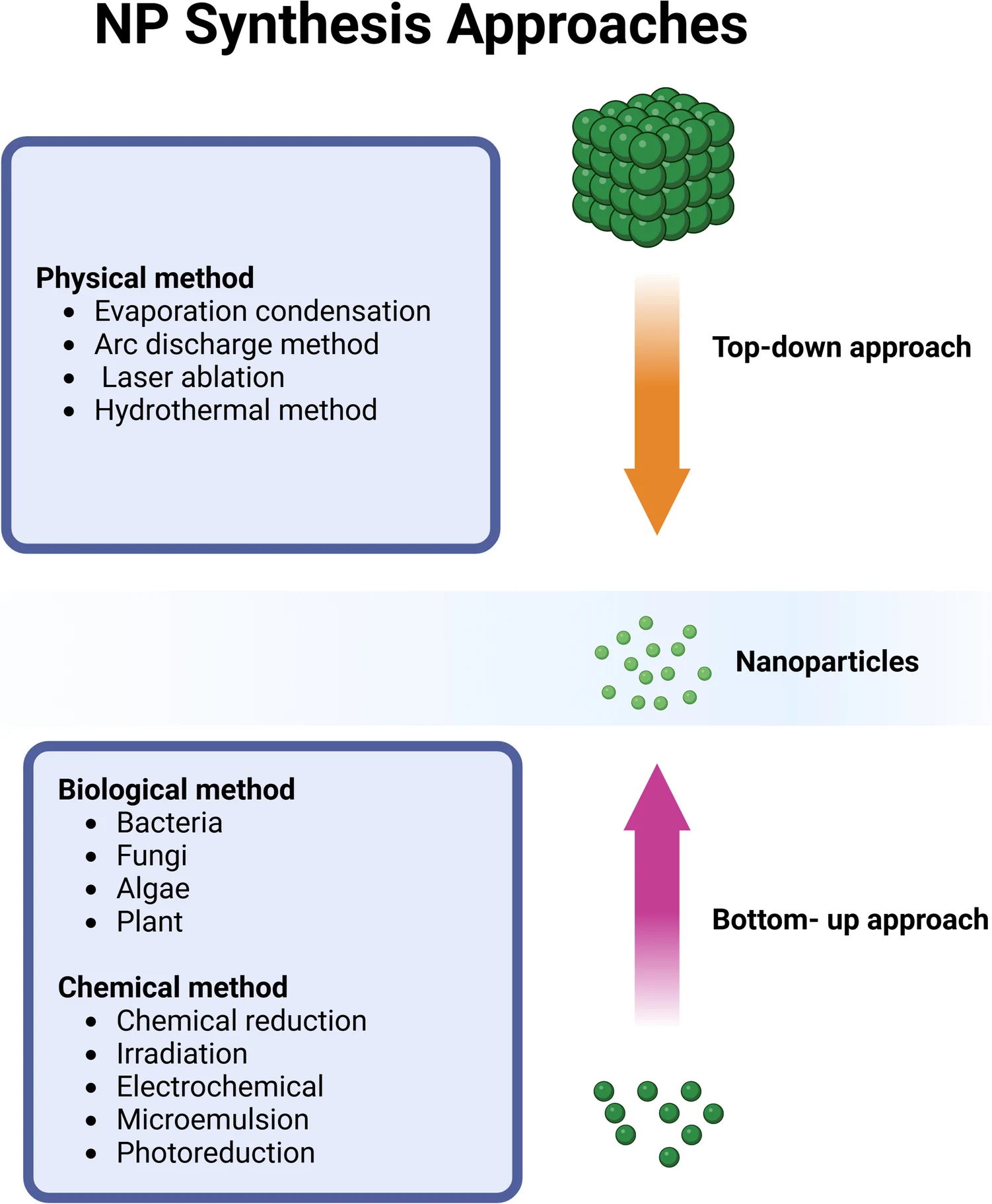
Protocols employed for synthesis of NPs bottom to top approach and top to bottom approach. Figure abridged from Ramanathan et al. (2021)

Physical, chemical, and biological methodologies can be adeptly employed in both top-down and bottom-up approaches, as depicted in Fig. 2 (Ramanathan et al. 2021). The top-down approach involves the transformation of bulk materials into smaller NPs. This strategy typically aligns with the domain of physical methods, requiring specialized equipment such as tube furnaces to reduce bulk materials into smaller particles. In contrast, the bottom-up approach is characterized by synthesizing NPs from molecular-level precursors. Chemical reduction methods frequently employ this approach, sometimes with capping agents to ensure NP stability. The biological method for NP synthesis is classified under the bottom-up paradigm, wherein biomolecules engage with metallic substances to produce nanoscale materials (Ramanathan et al. 2021).

### Biological synthesis of NPs

Certain microorganisms can generate metal NPs when exposed to metal ions, triggering a defensive mechanism that reduces these ions through redox enzymes, resulting in intra- or extracellular NP production (Mohd Yusof et al. 2020a). While primarily metal NPs are synthesized, some salts and metal oxides are also documented (Lahiri et al. 2021). Various microorganisms have been used for biogenic synthesis, including silver (Vijayakumar et al. 2023) and gold (Rajasekar et al. 2020). NPs can also form through biosorption, a passive process involving binding and ion exchange (Muñoz et al. 2021). The interaction between metals and bacterial cell walls is mediated by functional groups (Mrvčić et al. 2009).

Biological synthesis of NPs has gained interest for its sustainable, non-toxic, and reliable methods (Loi et al. 2023). Natural substances from plants and algae, as well as biomolecules from living cells, are used to produce nutritionally relevant NPs (Alsaiari et al. 2023; Maeh et al. 2019). Microorganisms are particularly valuable due to their ability to synthesize ZnO NPs with precise control over size and morphology (Bharathi et al. 2020). One of the critical advantages of biogenic NP synthesis lies in its obviating the need for reducing and capping agents, as the functional groups inherent in biological systems are responsible for governing NP nucleation and preventing aggregation (Sidhu et al. 2022). Specifically, microorganisms’ biogenic production of NPs stands out as a highly promising domain due to the rapid growth, cost-efficiency, and straightforward cultivation of microorganisms, along with the ease of controlling and manipulating their growth conditions and environment (Bahrulolum et al. 2021). Microbial NPs synthesis presents a distinct advantage over plant-based NP production which relies on extracting water soluble bioactive compounds, including polyphenols, saponins, organic acids, vitamins, and polysaccharides, using specific solvents such as methanol and acetone (Babitha and Korrapati 2013).

In summary, biogenic synthesis of NPs via microorganisms presents a promising avenue for environmentally sustainable and cost-effective production. This approach offers adaptability across various fields, including medical and food sciences. Further research and development in this area holds great promise for addressing current challenges in NP synthesis and expanding the possibilities for innovative applications.

### Biological synthesis of ZnO NPs using probiotic bacteria

The intrinsic attributes of probiotic bacteria render them compelling candidates for both laboratory and industrial-scale production of metallic and non-metallic NPs. Supported by the presumption of qualified safety (QPS by EFSA) and GRAS by the FDA, particularly in the case of LAB, these microorganisms are positioned as highly favorable biological platforms for NP synthesis (Harandi et al. 2021).

In a typical experiment, cultures are first grown. Then, metal precursors are introduced in the form of soluble salts. These salts are precipitated in a suspension that contains microbial cells or extracts. These extracts are derived from the biological compounds of the culture. The synthesis reaction is usually complete within a relatively short time frame, ranging from minutes to a few hours, contingent upon the specific conditions of the culture. The reaction typically manifests as the formation of white sediment at the bottom of the reaction vessels or discernible alterations in the hue of the suspensions and are reliable indicators of a successful transformation (Al-Tameemi et al. 2023b; Mahdi et al. 2021).

Not all microorganisms can synthesize NPs due to variations in their metabolic processes and enzymatic activities. Therefore, the selection of suitable microbial candidates is critical for the production of their specific enzyme activities and biochemical pathways (Mohd Yusof et al. 2019). Wastewater harbors bacteria with elevated tolerance to toxic substances and enzymatic reduction capabilities (Bhakta et al. 2012) and is therefore potentially rich in species proficient in NP synthesis (Allam et al. 2019). Due to their gram-positive classification, lactic acid bacteria (LAB) possess a substantial cell wall composed of peptidoglycan, lipoteichoic acid, proteins, polysaccharides, and (EPS) extracellular polymeric substances. These structural elements serve as sites for biosorption and/or the bioreduction of metal ions, owing to their negative electrokinetic potential, which attracts metal cations and initiates the synthesis of NPs (Król et al. 2018; Tiquia-Arashiro 2018). Additionally, this cell wall structure serves as a protective mechanism against the stresses induced by metallic substances (Mohd Yusof et al. 2020b).Various strains of LAB can produce and release EPSs and EPS. These compounds can serve as stabilizing agents by safeguarding cells against metal ions and offering additional sites for the biosorption of metal ions (Zeng et al. 2020; Mohd Yusof et al. 2019).

In this context, several bacterial species have been explored for their potential in NP synthesis, including representatives from the *Lactobacillus* and *Bacillus* genera as listed in Table 1. Notably, *L. plantarum* cell biomass and supernatant rapidly produced ZnO NPs, with sizes falling within the range of 349 nm and 351 nm (Mohd Yusof et al. 2020a). Other examples include *L. lactis*, *Bacillus* species (Mahdi et al. 2021), *Bacillus subtilis* ZBP4 (Hamk et al. 2023), *B. licheniformis* MTCC9555 (Tripathi et al. 2014), *L. johnsonii* (Al-Zahrani et al. 2018), *L. sporogenes* (Mishra et al. 2013), *L. paracasei* LB3 (Król et al. 2018), *L. gasseri* (El-Sayed et al. 2021), *W. confusa* UPM22MT04 (Al-Tameemi et al. 2023a), and *W. cibaria* UPM22MT06 (Al-Tameemi et al. 2023b), all of which demonstrate the capacity to synthesize ZnO NPs.

**Table 1:** Probiotic bacteria used for the synthesis of ZnO NPs

Probiotic bacteria	Synthesis	Size (nm)	Morphology	Applications	References
*L. plantarum* TA4	Extracellular and intracellular	349 and 351	Flower	Antibacterial	Mohd Yusof et al. (2020a)
*L. lactis* NCDO1281(T) and *Bacillus* sp PTCC 1538	Intracellular	99 and 55–61	Nano-rods and spheres	Electrochemical determination of bisphenol A	Mahdi et al. (2021)
*B. subtilis* ZBP4	Extracellular	22–59	Irregular spherical	Antibacterial	Hamk et al. (2023)
*B. licheniformis* MTCC9555	Intracellular	250 to 1 µm	Flower	Photocatalytic	Tripathi et al. (2014)
*L. sporogens*	Intracellular	146	Hexagonal	Antibacterial	Mishra et al. (2013)
*L. paracasei* LB3	Intracellular	1197	-	-	Król et al. (2018)
*L. gasseri*	Extracellular	282	Hexagonal	Antimicrobial	El-Sayed et al. (2021)
*B.* haynesii MG822851	Extracellular	50	-	Antibacterial	Rehman et al. (2019)
*L. plantarum* ZDY2013	Extracellular	41	Spherical	Antibacterial	Li et al. (2021)
*L. johnsonii*	Intracellular	4–9	Spherical	-	Al-Zahrani et al. (2018)
*W. confusa UPM22MT04*	Extracellular	2–8	Spherical	Antibacterial	Al-Tameemi et al. (2023a)
*W. cibaria UPM22MT06*	Extracellular	2–8	Spherical	Antibacterial	Al-Tameemi et al. (2023b)

Comparative analysis with prior investigations highlights superior small size of the synthesized ZnO NPs (Table 1). Previous research utilized various microorganisms or methods for ZnO NP synthesis. Significantly, Al-Tameemi et al. (2023b) reported on the synthesis of ZnO NPs that are considerably smaller, with greater potential in biological applications. The observed synthesis of ZnO NPs, characterized by minimal accumulation, may be attributed to the wastewater strain’s capability to resist zinc nitrate, its secretion of specific biological substances that modulate NP nucleus growth and prevent aggregation, and the employed biological synthesis technique (Al-Tameemi et al. 2023a). Further investigations are warranted in the application of ZnO NPs with enhanced properties, potentially leading to applications in the development of antibacterial agents and other fields.

Available evidence supports the capability of probiotic bacteria to produce ZnO NPs, indicating the importance of further exploration into additional potential probiotic strains for this purpose. This innovative approach of employing probiotic bacteria for the biological synthesis of ZnO NPs not only broadens the scope of potential biotechnological applications but also advances our understanding of eco-friendly NP synthesis.

### Mechanism of intracellular synthesis of ZnO NPs by microorganisms

The intracellular synthesis pathway of ZnO NPs is governed by the microbial cell wall structure and its ionic charges. In this process, zinc ions are directed into the cell, where they amalgamate with various molecules, including enzymes and coenzymes, culminating in the formation of ZnO NPs (Slavin et al. 2017).

Under metal stress, the bacterial cell wall acts as a biosorption site. Its negatively charge functional groups (carboxyl, phosphate, and hydroxyl) attract the positively charged metal ions through electrostatic interactions (Tiquia-Arashiro 2018). Following this, the ions are absorbed by the bacterial cells. The entrapped metal ions are then reduced to their atomic form. This reduction occurs because NADH provides electrons to the ions. The enzyme that facilitates this electron transfer, known as NADH-dependent reductase, is located in the plasma membrane. Finally, the resulting nuclei transform into NPs, which accumulate within the cytoplasm or the periplasmic region (Altammar 2023; Slavin et al. 2017). These NPs are capped and stabilized by the peptides, proteins, and amino acids such as tyrosine, cysteine, and tryptophan present in the cells (Balraj et al. 2017).

**Fig. 3:**
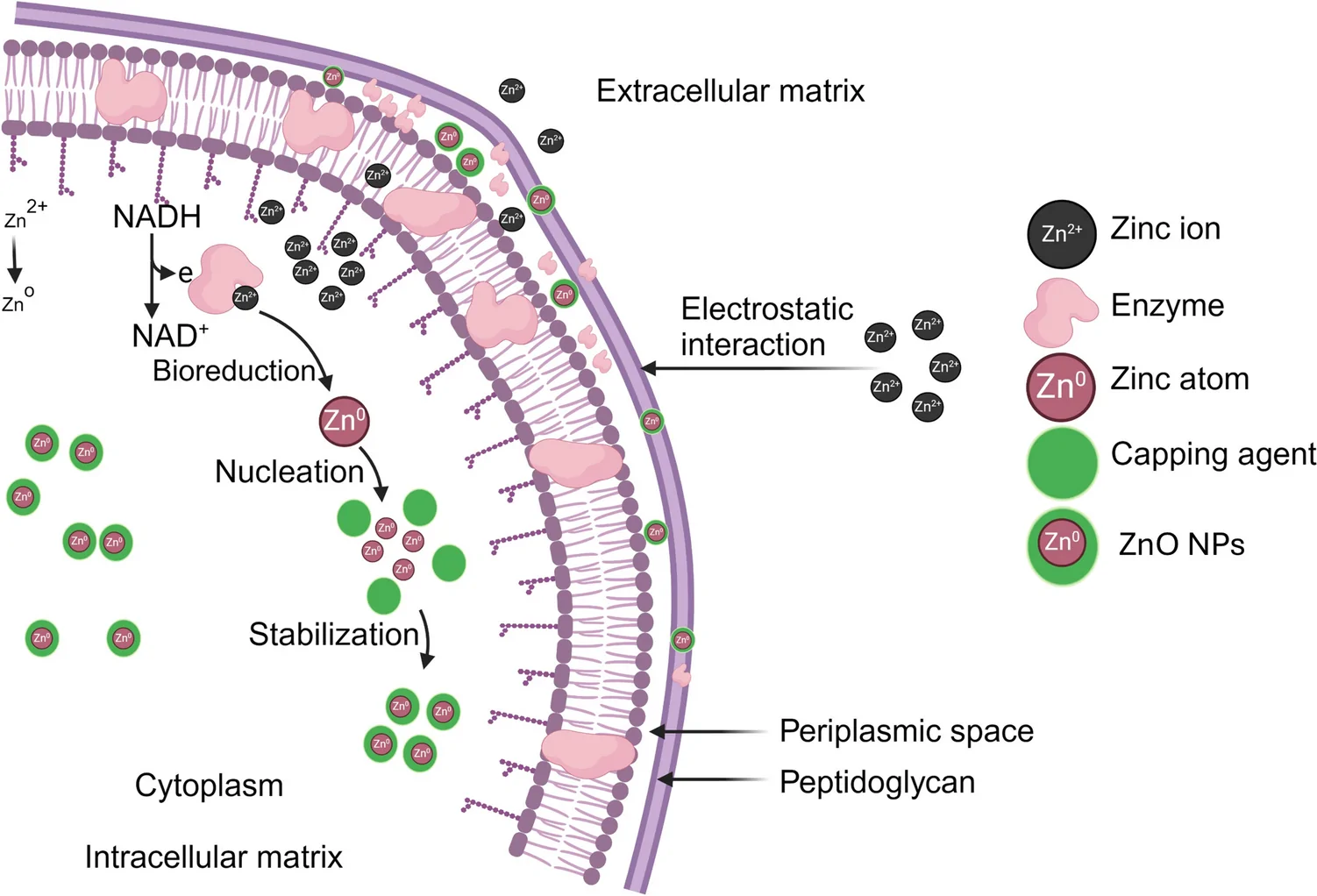
The intracellular synthesis mechanisms of ZnO NP formation. Metal ions are electrostatically transported across the cell wall. NPs form in the periplasmic space and cytoplasm after the reduction of metal ions to metal atoms by enzymes (e.g., NADH cofactor and NADH-dependent reductase). The peptides or protein and amino acids such as tyrosine, cysteine, and tryptophan are responsible for promoting NP stabilization. Figure abridged from Mohd Yusof et al. (2019)

The process is similar during the formation of ZnO NPs. The electrostatic transference of zinc ions into the cell results in the reduction of these ions (Zn^2+^) to zinc atoms (Zn^0^) by cell wall enzymes. This reduction process expands the nuclei, forming ZnO NPs within the periplasm or cytoplasm (Fig. 3) (Tripathi et al. 2014; Król et al. 2018). Ultrasonication is necessary to break open the cells and isolate intracellular NPs by disrupting the cell membranes and releasing the NPs into the solution. During ZnO synthesis, the probiotic species, *Lactobacillus sporogens*, uses membrane-bound oxidoreductases that are active at low pH. This suggests that low pH is required for ZnO NPs synthesis. It could also be the case that the creation of ZnO NPs in *L. plantarum* culture solution results from the carbon source-dependent rH2 and pH-sensitive oxidoreductases present in this species (Selvarajan and Mohanasrinivasan 2013).

### Mechanism of extracellular synthesis of ZnO NPs by microorganisms

**Fig. 4:**
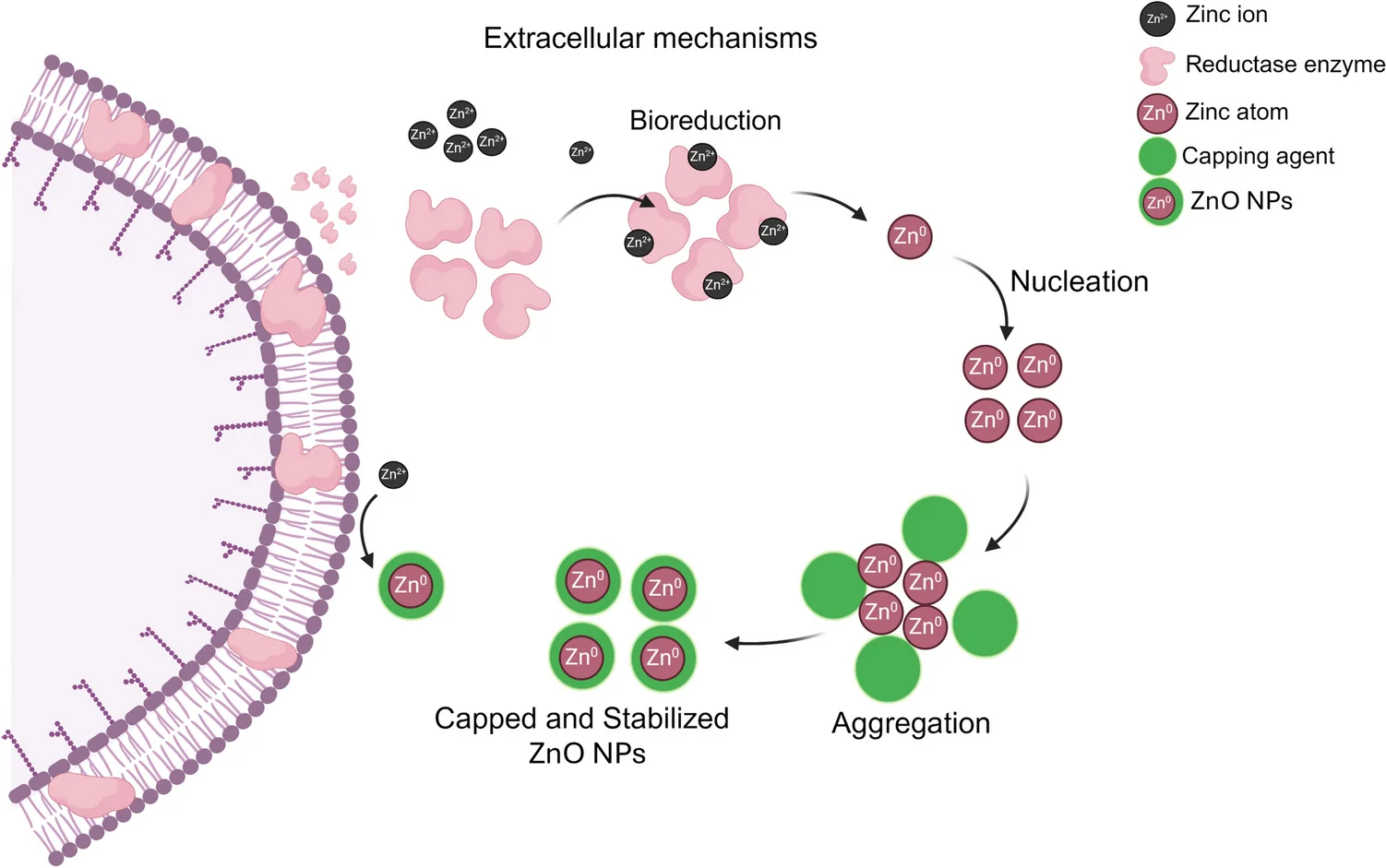
Generalized mechanism of microbial extracellular synthesis of ZnO NPs. Extracellular processes involve enzymatic mediation, notably through the action of nitrate reductase enzymes, which are excreted into the growth medium. These enzymes facilitate the reduction of metal ions to their corresponding metal atoms, initiating nucleation and subsequent NP growth. Functional groups of bacterial protein such as amine, carboxyl, and hydroxyl in supernatant act as capping agents by offering stability to NPs synthesized. The manifestation of white precipitation in the medium serves as a visual indicator of NP production. Figure abridged from Mohd Yusof et al. (2019)

Unlike intracellular synthesis, extracellular synthesis involves enzymatic processes occurring either on the microbial cell membrane or through the release of enzymes, such as cofactor NADH and NADH-dependent enzymes, into the growth medium. After NADH electrons are transferred through NADH-dependent reductase (such as the nitrate reductase enzyme), Zn^2+^ is reduced to Zn^0^, forming ZnO NPs (El-Belely et al. 2021; Kundu et al. 2014). In a study conducted by Durán et al. (2005), it was confirmed by nitrate reductase assay that NADH-dependent reductase can serve as a reducing agent for silver nitrate, facilitating the formation of silver NPs. Kundu et al. (2014) investigated the role of secreted enzymes or proteins in ZnO NP synthesis by heavy metal resistant-*Rhodococcus pyridinivorans* NT2. They exposed the bacterial biomass to zinc ion and used distilled water as a control. The protein expression profile indicated that extracellular protein was secreted at twice the level when zinc ions are present (1113 ± 6.3 µg/mL^−1^) compared to the control (554 µg/mL^−1^). Furthermore, SDS-PAGE showed a protein with a molecular mass of 43 kDa, indicating it was the right size for NADH-dependent reductase. While NADH-dependent reductases are likely contributors to NP synthesis, further work is required to definitively identify the enzymes involved. Figure 4 visually represents the extracellular synthesis mechanism of ZnO NPs mediated by microbes.

### Mechanism of NP synthesis by metal-resistant bacteria

It is hypothesized that a bacterium’s tolerance for metal ions underpins its ability to synthesize metal NPs. Bacteria can reduce metal ions to nanoscale metal particles through metal ion biosorption and bioaccumulation. This process depends on the presence of functional groups on the cell walls, enzymes, and coenzymes within the cell to reduce metals ions and form NPs in the cytoplasm or periplasmic space (Mohd Yusof et al. 2020b).

Metal tolerance arises in bacteria from their need to survive and grow under conditions of metal stress. They employ several mechanisms to achieve this, including intracellular sequestration, active efflux, enzymatic transformation, and oxido-reduction of metal ions (Zeng et al. 2020). In addition to these mechanisms, certain bacteria, such as *Bacillus* sp. S3, can produce extracellular polymeric substances (EPS) and proteins when exposed to heavy metal toxicity. This results in the precipitation of heavy metal ions onto the cell surfaces facilitated by the negatively charged functional groups of EPS. This process reduces heavy metal toxicity and the EPS serves as a protective barrier, protecting the cells in toxic environments (Zeng et al. 2020). EPS can be located on the cell surface or in the surrounding environment (Zeng et al. 2020). Heavy metals bind to EPS through various mechanisms including complexation, surface adsorption, precipitation, and ion exchange (Priyadarshanee and Das 2023).

Several studies have shown that probiotic LAB such as *P. pentosaceus* M132-2, *L. sakei* M129-1, and *W. paramesenteroides* MYPS5 can form biofilms that produce EPS (Kim et al. 2022; Yadav and Sunita 2022). Biofilm is a surface-adhesive community of bacteria that produces EPS (Jayathilake et al. 2017). Biofilm-EPS composed mainly of polysaccharides (e.g., homopolysaccharides such as α-D-glucans, β-D-glucans, and fructans; heteropolysaccharides such as pyruvate, succinate, and formate), negatively charged functional groups such as hydroxyl and carboxyl groups, proteins such as glycoproteins, extracellular enzymes (e.g., hydrolase and oxidoreductase), and uronic acids as major components, with nucleic acids and lipids as minor constituents (Wei et al. 2023).

**Fig. 5:**
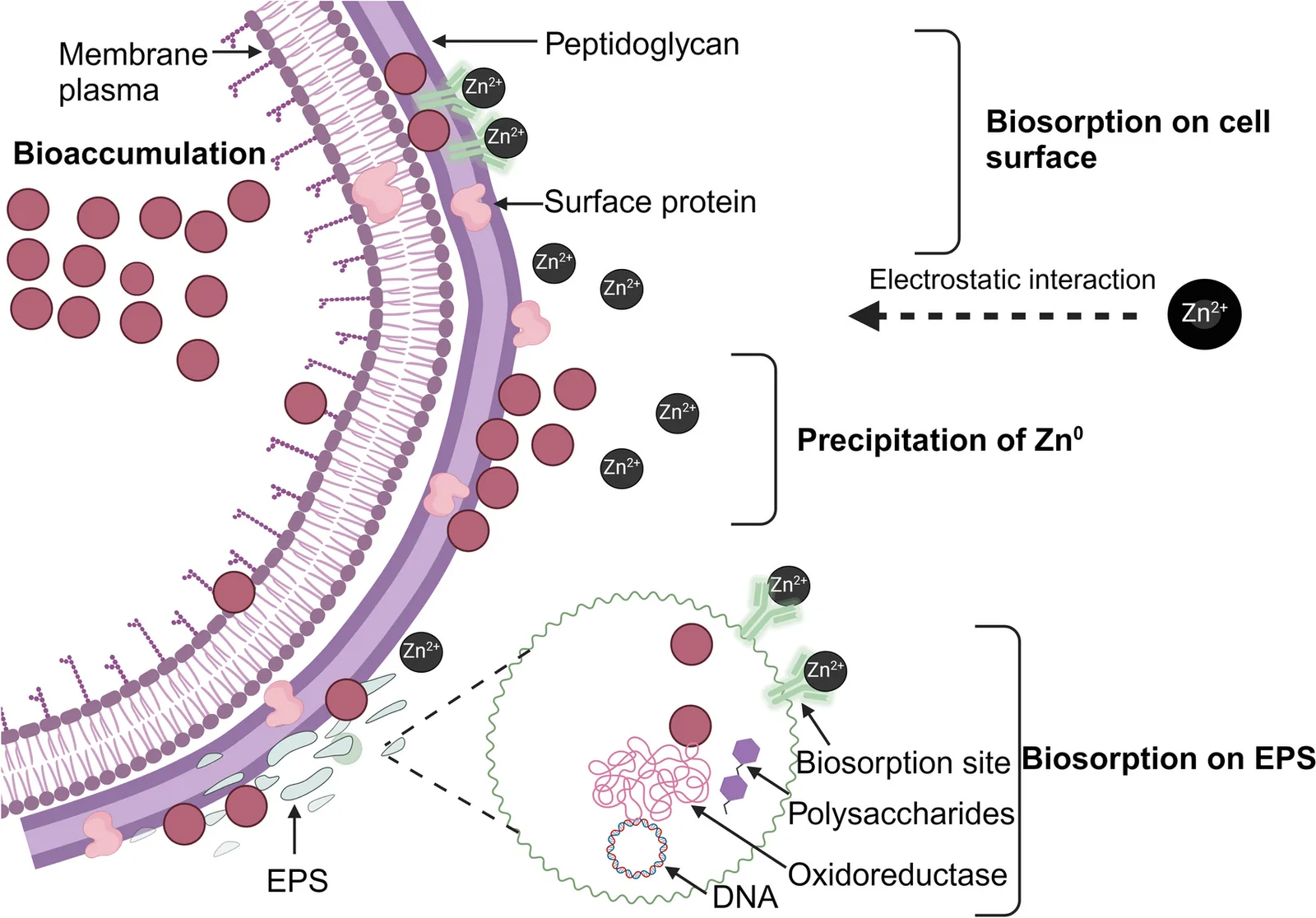
A Gram-positive bacteria’s mechanism for resisting metal ions and reducing them to their respective metal NPs. The bacterial cell wall acts as a biosorption site when its negative charge functional groups (carboxyl, phosphate, and hydroxyl) bind with metal ions. Biofilms-EPS secreted by bacteria can also act as biosorption sites, by trapping metal ions in the EPS matrix and potentially converting them into less toxic forms. Precipitation, a reaction between anions, such as hydroxyl ions and metal ions (cations), also reduces the toxicity of metal ions. This reaction forms solid particles and can happen inside or outside the cell. Bioaccumulation, unlike biosorption, is an active procedure that requires energy. It involves the uptake and binding of metal ions inside the cell. Low-molecular-weight proteins, such as metallothioneins, help in this process by binding to the metal ions and facilitating their accumulation within the cell. Figure abridged from Mohd Yusof et al. (2020b) and Wei et al. (2023)

Biofilms formed from EPS secreted by bacteria can also act as biosorption sites, by capturing metal ions in the EPS matrix and potentially converting them into less toxic forms (Tiquia-Arashiro 2018). Moreover, these EPS contribute to NP synthesis through secretory of protein and polysaccharide that act as reducing and stabilizing agents (Ran et al. 2024). EPS-functional groups (e.g., carboxyl, amide, and hydroxyl) act as capping and stabilizing reagents for synthesized NPs (Ran et al. 2024). Figure 5 shows a Gram-positive bacteria’s mechanism for resisting metal ions and reducing them to their respective metal NPs.

### Bacterial extract as a capping agent in NP synthesis

Bacterial extracts can be used to synthesize NPs in the form of a reduction agent and a stabilizing agent. Probiotic LAB such as *L. plantarum*, *P. acidilactici*, and *W. cibaria* PN3 can release biosurfactants (Yan et al. 2019; Subsanguan et al. 2020). A biosurfactant is an amphiphilic molecule that plays an important role in NP stabilization and helps the NP to preserve a regular shape (Kamalesh 2024) and prevent them from aggregating. This stabilization of NP by biosurfactants occurs through various mechanisms, including electrostatic interactions, hydrogen bonding, and hydrophobic interactions between the biosurfactant molecules and the NP surface (Kamalesh 2024). Synthesis of biosurfactant-based silver and gold NPs with purified lipopeptide were isolated from *B. subtilis* ANR 88. The gold NPs were mostly hexagonal and ranged from 40 to 60 nm, while the silver NPs were spherical and sized between 4 and 18 nm. The lipopeptide helped stabilize the NPs and allowed their synthesis without needing chemical-reducing agents, making it a promising option for eco-friendly NP production (Rane et al. 2017). In addition, several studies reported that probiotic LAB such as *L. plantarum*, *W. cibaria* NC516.11, and *L. brevis* L010 can also produce and release the EPSs (Silva et al. 2019; Li et al. 2022; Kwun et al. 2024). EPSs are high molecular weight carbohydrate polymers and are secreted by microbes (Dey et al. 2023). Bacterial-EPSs reduces metal ions and stabilizes NPs using EPS functional groups like carboxylic, phosphate, sulfate, and hydroxyl (Dey et al. 2023). Ran et al. (2024) investigated secreted bacterial EPS for stabilizing NPs synthesis. They exposed the selenium NPs (SeNPs) to EPS and SeNPs without EPS as a control. Dynamic light scattering (DLS) was used to determine the size of NPs. The DLS results showed that the size of SeNPs without EPS was 1322.7 nm, unstable and rapidly aggregated, while the size of SeNPs-EPS was 1177.2 nm and stable. The findings showed that EPS secreted from bacteria resulted in the stabilization of NPs. Furthermore, proteins and carbohydrates play an important role in physicochemical processes like biosorption, complexation, nucleation, growth, and stabilization (Lahiri et al. 2021). In summary, bacterial extracts, along with biosurfactants, serve as both reducing and stabilizing agents in NP synthesis, facilitating eco-friendly production. Additionally, biomolecules such as proteins, carbohydrates, EPS, and bacterial EPSs contribute to NP stabilization through various mechanisms, ensuring stability and offering a promising avenue for efficient NP synthesis with diverse applications.

## Biomedical applications of ZnO NPs

**Fig. 6:**
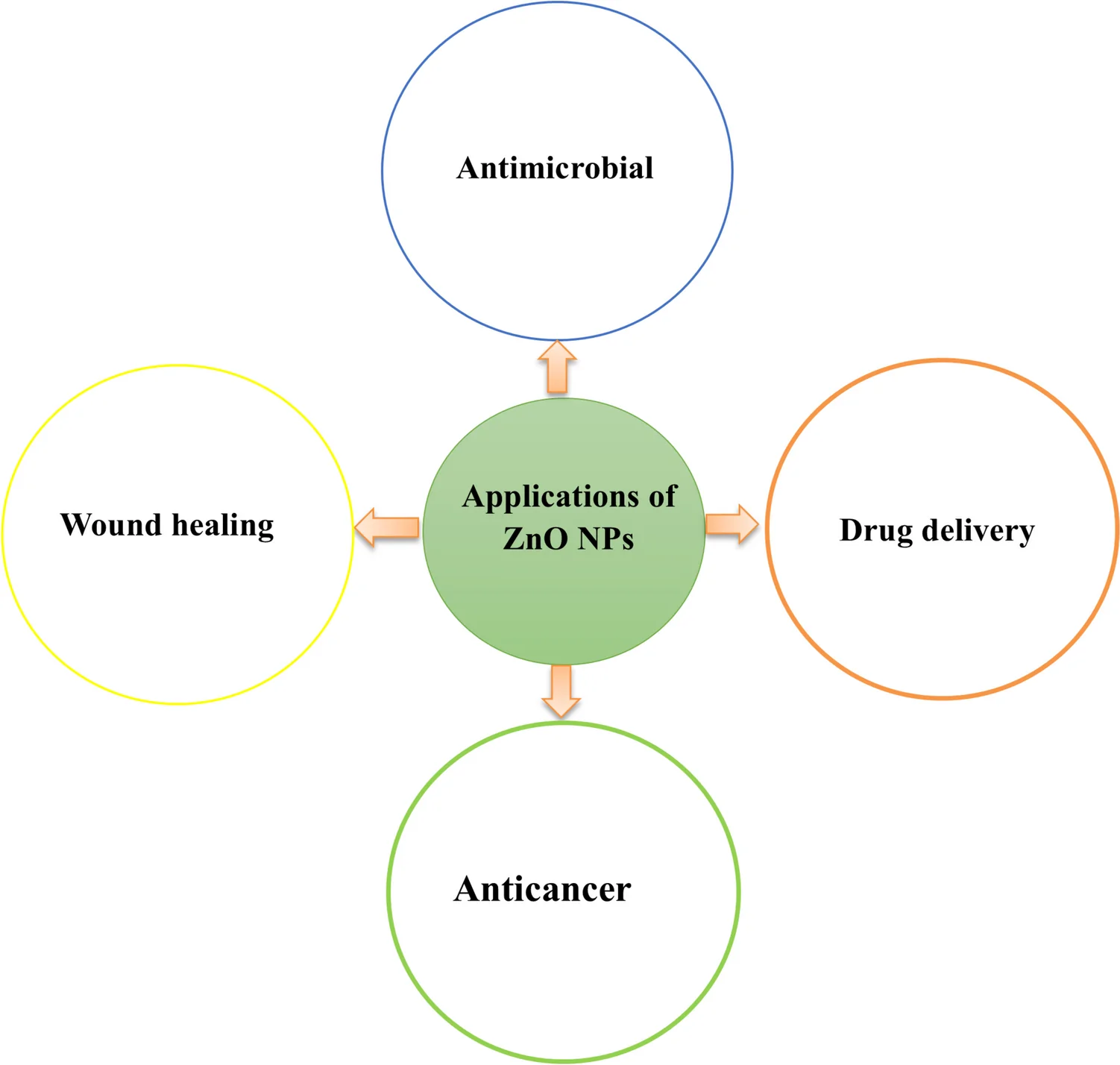
Applications of ZnO NPs

ZnO NPs, being cost-effective and low-toxic, have gained significant attention in biomedical fields due to their antimicrobial, anti-inflammatory, wound-healing, and anticancer properties (Kim et al. 2017) (Fig. 6).

### Antimicrobial activity of ZnO NPs

The rise of multidrug-resistant microbes has necessitated the development of new effective techniques to combat infectious diseases. Nanomaterials such as ZnO NPs have emerged as a novel solution (Al-Tameemi et al. 2023b). NPs exhibit antimicrobial properties, owed to their high reactivity due to the large surface area to volume ratio, morphology, porosity, and composition that allow them to interact easily with microorganisms’ surfaces (Batool et al. 2021).

Several investigations have demonstrated the antibacterial and antifungal properties of ZnO NPs against many bacteria and fungi, encompassing gram-positive and gram-negative strains (Jain et al. 2020; Mohamed et al. 2021). ZnO NPs synthesized by *B. haynesii* have minimal inhibitory concentrations (MIC) and minimal bactericidal concentrations (MBC) of 4 and 8 mg/mL for *S. aureus* and 8 and 16 mg/mL for *E. coli*, respectively (Rehman et al. 2019). Specifically for Gram-positive bacteria, the MIC values for ZnO NPs were measured at 0.625 mg/mL. They are active against Gram-positive bacteria like *S. aureus*, including its drug-resistant variant MRSA (Al-Tameemi et al. 2023a). Furthermore, ZnO NPs have been shown to have anti-virulence and anti-biofilm properties. In a study by Valadbeigi et al. (2023), ZnO NPs showed excellent biofilm-destroying properties on biofilms formed by the *P. aeruginosa*.

ZnO NPs have promising antifungal properties against a variety of harmful fungi and yeasts. Biosynthesized ZnO NPs show significant antifungal activity against *F. solani*, *F. oxysporum*, *S. sclerotia*, and *A. terreus* (Mohamed et al. 2021). Furthermore, ZnO NPs synthesized by *Serratia nematodiphila* showed good antifungal activity against *Alternaria* sp. and *Xanthomonas oryzae* pv. *oryzae* (Jain et al. 2020).

ZnO NPs demonstrate impressive antimicrobial potential due to their unique properties. They effectively combat many bacteria, including drug-resistant strains, and are proficient at reducing biofilms and pathogen virulence. Their antifungal properties are promising, particularly for skin infections.

### Anticancer activity

Microbially synthesized ZnO NPs show significant anticancer properties and serve as effective drug delivery carriers due to their biocompatibility and low cytotoxicity (Kundu et al. 2014; Sholkamy et al. 2024). They enable targeted delivery of anticancer agents to tumor cells, minimizing damage to healthy tissues. For example, ZnO NPs loaded with anthraquinone selectively target HT-29 cancer cells (Kundu et al. 2014). They also demonstrate effectiveness against MCF-7 and HepG-2 cancer cell lines (Abdelhakim et al. 2020) and show significant toxicity against HT-29 cells (Suba et al. 2021). These findings suggest that microorganism-mediated ZnO NPs may be essential in long-term anticancer therapy by providing precise and biologically safe drug delivery vehicles.

### Wound healing

Microbial infections, particularly those caused by microorganisms like MRSA, often impede wound healing (Oliva et al. 2023). Metal oxide NPs, including ZnO NPs, possess intrinsic antimicrobial properties and can expedite the wound-healing process (Asif et al. 2023). It has been demonstrated that ZnO NPs help to promote wound healing in several studies. For instance, a gel containing ZnO NPs was found to be an efficient topical antimicrobial and wound-healing agent in rats (Shao et al. 2018). Also, cotton wound bandages containing ZnO NPs showed antimicrobial properties that were suitable for the treatment of infection-prone wounds, such as those caused by diabetes or burns (Khatami et al. 2018).

It is essential that ZnO NPs are applied at the right dosage and the appropriate duration (Ezealisiji et al. 2019). Wound healing, the immune response, and inflammation are intimately interconnected such that ZnO NPs aid in the re-epithelization of the skin via an anti-inflammatory influence, by suppressing the expression of inflammatory marker genes such as IL-6 and TNF-α (Han et al. 2023). Zinc also plays a pivotal role in enhancing platelet activity and aggregation, thus supporting the wound healing process (Sekhon and Sen Gupta 2017). Moreover, zinc supplementation boosts immunity and reduces chronic harmful inflammation (Hojyo 2016). Another hallmark effect of zinc is the enhancement of human dermal fibroblast migration mediated by reactive oxygen species (Lin et al. 2017). In addition, zinc deficiency can also impair wound healing, making ZnO NPs an attractive therapeutic alternative for improving wound healing (Lin et al. 2017).

### Antibacterial mechanisms of ZnO NPs

ZnO NPs exhibit antimicrobial activity by disrupting plasma membrane permeability and releasing Zn^2^⁺ ions, which inhibit active transport and induce oxidative stress through reactive oxygen species (ROS) generation, leading to cell death (Mendes et al. 2022). Their positive zeta potential enhances attachment to negatively charged microbial cells, causing membrane damage and leakage of intracellular contents (Gomaa 2022).

**Fig. 7:**
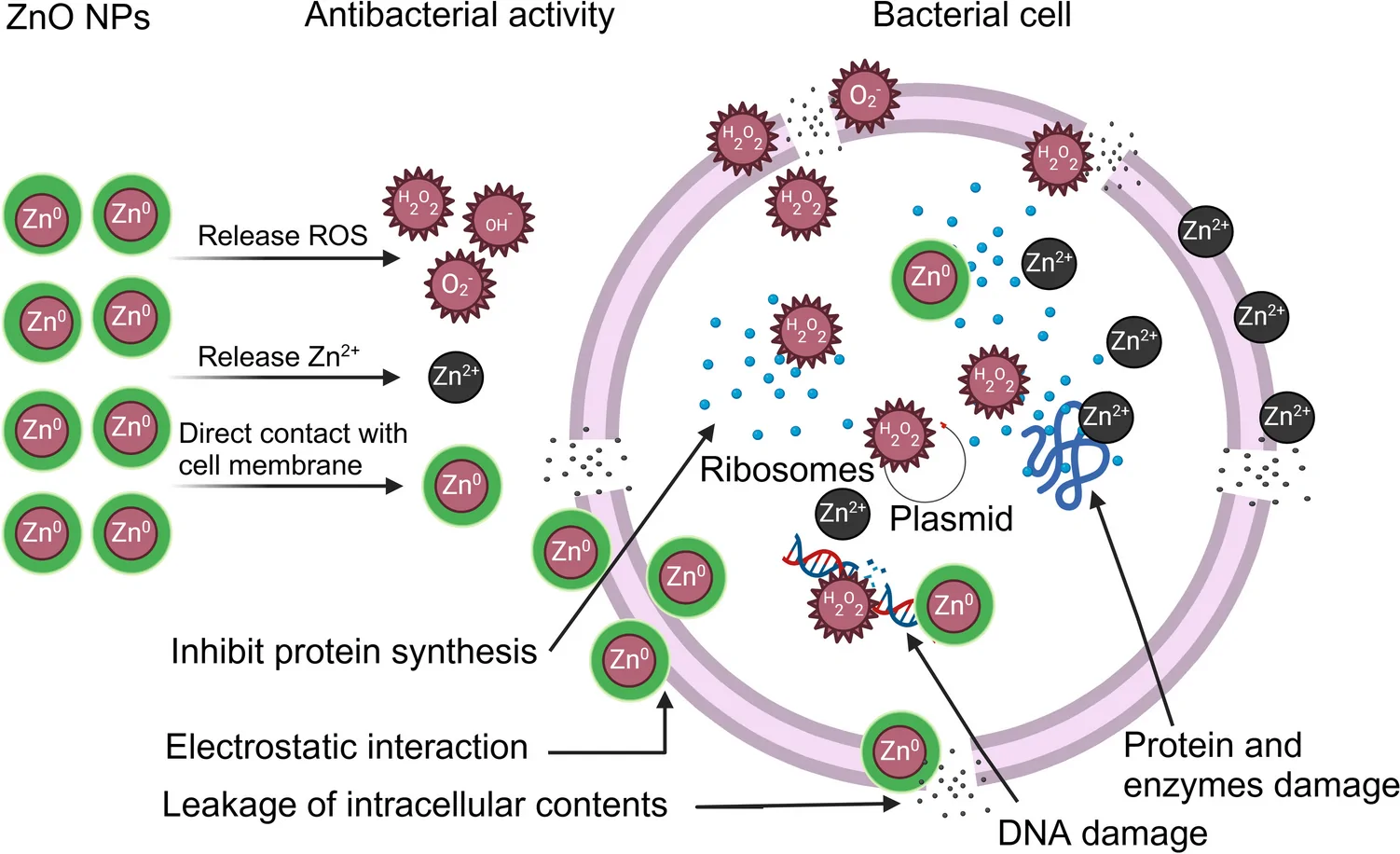
The antibacterial mechanisms of ZnO NPs against pathogenic bacterial cells. Figure abridged from Dimapilis et al. (2018)

ZnO NPs also bind to components in the cell wall of *Streptococcus pyogenes*, resulting in cell wall disruption (Lianga et al. 2020). The release of Zn^2^⁺ ions disrupts the phospholipid bilayer and causes the loss of cytoplasmic constituents (Soren et al. 2018). Additionally, Zn^2^⁺ ions interact with thiol groups in respiratory enzymes, generating ROS that damage cellular structures and mitochondrial functions (Fontecha-Umaña et al. 2020). Figure 7 shows the antibacterial mechanisms of ZnO NPs against pathogenic bacterial cells.

## Factors affecting the antimicrobial activity of ZnO NPs

The antimicrobial activity of ZnO NPs is dependent on its shape, size, concentration, and microbial species.

### Morphology of ZnO NPs

Different morphologies of ZnO NPs, such as flower, rod, and pyramid shapes, exhibit varying levels of antimicrobial activity. Sharp-edged NPs generally show superior effects due to their ability to penetrate microbial cell walls (Saif et al. 2019; Talebian et al. 2013).

### Size of ZnO NPs

The particle size of ZnO NPs corresponds to increased antimicrobial activity. This can be attributed to their larger surface area-to-volume ratio, allowing them to bind more ligands (Agarwal et al. 2019). Particles around 12 nm inhibited the growth of *S. aureus* more effectively than those exceeding 100 nm (Jones et al. 2008). Specifically, smaller ZnO NPs with a larger surface area exhibited increased oxygen species generation and consequently increased hydrogen peroxide production.

### Concentration of ZnO NPs

The antimicrobial activity of ZnO NPs increases with their concentration (Thirumoorthy et al. 2021). This dose-dependent relationship has been observed against a spectrum of microorganisms, including *S. aureus*, *B. subtilis*, *P. aeruginosa*, *P. mirabilis*, *E. coli*, *C. albicans*, and *C. tropicalis* (Elumalai and Velmurugan 2015), and resulted in significant antibacterial, antifungal, and antioxidant activities (Rajeshkumar et al. 2021). ZnO NPs synthesized by *Xylaria acuta* also exhibited dose-dependent antimicrobial and anticancer activities (Sumanth et al. 2020). These findings highlight the crucial role of ZnO NP concentration in enhancing antimicrobial and therapeutic potential.

### Species of microorganism

The microbial species to be treated is an essential factor affecting the antimicrobial properties of ZnO NPs. ZnO NPs are most effective against *B. subtilis* relative to other strains such as *P. aeruginosa* and *E. coli* (Azam et al. 2012). Similarly, *S aureus* shows heightened susceptibility in comparison to various fungal and bacterial strains, including *C. tropicalis* and *C. albicans*, *E. coli*, *B. subtilis*, *P. mirabilis*, and *P. aeruginosa* (Elumalai and Velmurugan 2015). *S. aureus* is more sensitive to ZnO NPs synthesized using *L. plantarum* TA4 than *E. coli* (Mohd Yusof et al. 2021). Gram-positive bacteria, cell walls rich in peptidoglycan, teichoic acid, and ample pores are more sensitive to ZnO NPs than Gram-negative bacteria which have cell walls rich in lipopolysaccharides and lipoproteins that act as a barrier to NP entry (Naseem and Durrani 2021).

### Pathogenic bacteria causing skin infections as a case for NP treatment

Skin infections caused by pathogenic bacteria, particularly *S. aureus* and *Streptococcus* species, are a significant concern (Allaw et al. 2023). MRSA, classified into healthcare-associated MRSA (HA-MRSA) and community-associated MRSA (CA-MRSA), complicates treatment due to its biofilm formation and virulence factors (Nikolic and Mudgil 2023). The skin is vulnerable to infections ranging from cellulitis to necrotizing fasciitis (Allaw et al. 2023). The rise of MRSA has led to increased complications and treatment failures, necessitating alternative therapies (Oliva et al. 2023). Recent studies indicate that NPs, like extracellular ZnO NPs, exhibit antibacterial properties against MRSA due to their release of free Zn2 + ions, particulate ZnO, and reactive oxygen species (Al-Tameemi et al. 2023b; Mendes et al. 2022).

## Conclusion

Microorganisms, especially probiotic and lactic acid bacteria, serve as eco-friendly nanofactories for synthesizing ZnO NPs. Their non-pathogenic nature and high enzyme production make them ideal for food and pharmaceutical applications. These bacteria can biosorb and bioreduce metal ions due to their unique cell wall structures and exopolysaccharides, allowing for a simple, safe, and scalable synthesis process without hazardous chemicals. Biologically produced ZnO NPs exhibit useful biomedical properties, including antimicrobial, anticancer, anti-inflammatory, and wound healing effects. Given the limited research in this area, exploring additional microbial candidates, particularly wastewater bacteria, could enhance the synthesis of ZnO NPs with desirable antibacterial properties.

## Data Availability

The data that supports this review are available in the published literature as referenced.
